# Quality of Breast Cancer Information on the Internet by African Organizations: An Appraisal

**DOI:** 10.1155/2017/2026979

**Published:** 2017-01-11

**Authors:** Cynthia Pomaa Akuoko

**Affiliations:** Christian Service University College, Kumasi, Ghana

## Abstract

*Objective*. The aim of this study was to appraise the quality of information on BC available at websites run by organizations in Africa.* Methods*. Three searches were conducted using Google search engine to generate a list of websites. The identified websites were assessed using European Commission (EC) quality criteria for health-related websites, which comprises different assessment areas including, completeness, transparency and honesty, authority, privacy and data protection, updating of information, accountability, and accessibility.* Results*. Thirteen (13) websites were included in the evaluation. Majority of the websites evaluated had low scores on the completeness and transparency of their websites. Scores on accessibility were however moderate and high for most of the websites. Breast cancer-specific organizations provided the highest quality information, particularly in terms of completeness. The overall lowest and highest quality scores were 9 and 43 out of 63, respectively, and 77% of the included websites scored less than 50% of the total quality score.* Conclusion*. This review has provided evidence of inadequate and inaccurate BC information provided by some cancer organizations in Africa. Considerable effort is required to make BC information on the Internet a valuable and up-to-date source for both professionals and patients.

## 1. Introduction

Education is important for both patients and their families [1]. It increases patients' knowledge about their health problems and offer treatment options, thus empowering them to make informed decisions about their health [2]. Patients however do not rely on a single source but use a combination of sources, which vary with time, in order to satisfy their information needs [3]. An investigation into potential psychological benefits for BC patients by surfing the Internet/World Wide Web (WWW) for medical information was conducted in Canada [4]. The researchers proposed that the Internet has potential to provide information about the specific type of cancer and validated treatment recommendations for patients. A study [5] on the reliability of health information on the Internet also suggested that the Internet is an important information resource that could improve patients' knowledge regarding their health problems. Furthermore, patients who use the Internet have significantly higher levels of understanding regarding their condition than those patients who do not and this empowers them to be much more involved in their treatment [6].

The Internet has become a powerful worldwide information source. The public has turned to the Internet, a WWW of interconnected computer networks for health information. Health information that was previously inaccessible is becoming freely accessible to increasing number of healthcare providers and consumers because of Internet use [7]. Millions of people are using the Internet from their homes, workplaces, and other places to obtain information about their health. In the USA, 55% of the online population turn to their Internet for health-related information to inform their decision-making process and health seeking behaviours [8]. The Internet has the potential to shape healthcare delivery and policies as well [9]. However, surfing the Internet for specific health-related information can be tedious and time-consuming despite all the search engines available to assist the search [7]. Judging the quality of and choosing appropriate resources to inform decision-making [10] are a challenge for Internet users. Internet users rely on a number of Internet resources (evaluation sites) that review and rate websites that provide health information to determine which sites to seek health-related information from in order to inform their decision-making [7].

It is acknowledged that cancer patients in general desire to know as much information about their condition as possible, be it good or bad [11]. Findings from a survey conducted in UK [12] suggested that most patients with cancer want as much information as possible about treatments and illness. A survey [13] identified that patients having access to a wide array of information makes them more comfortable or confident about their care. Such health-related information attained from the Internet provides the opportunity for patients to interact efficiently with healthcare professionals. However, this would depend on the quality of the information available to the consumer.

A systematic review was undertaken in 2002 [14], which assessed articles that investigated the quality of health information provided for consumers on the Internet. It reported that the quality of health information on the Internet could be poor and misleading. Previous studies also reported similar findings [15]. It was identified that much of the information is of poor quality and often inaccurate or misleading. Other studies undertaken to ascertain the quality of information provided on website on conditions such as gastrointestinal diseases, infertility, chronic pain, depression, obesity, childhood asthma, and coeliac disease scored poorly with regard to depth, accuracy, and reliability and also lacked information pertaining to funding sources and the date of the most recent update [16–23]. Most studies that specifically assessed the quality of websites with BC information also reported on gaps in the accuracy of the information [24–26].

Researchers have proposed that high quality websites should provide information that is complete, transparent, and user friendly (accessible) [17, 24, 27]. In terms of completeness the breadth and depth of the various topics such as risk factors, screening and mammography, various treatments, and breast reconstruction should be covered. Transparency, comprising the authorship and their credentials, attribution, currency, and disclosure of the information provided should be made clear. Accessibility of websites entails navigation through the site with easiness, having external scientific links to find specific information relating to BC, combination of audio, text, and visual format, colour coordination, and language.

In response to the need to increase awareness of BC in women, many nonprofit, nongovernmental, and governmental organizations have evolved to promote understanding of the disease. Healthcare workers in the developing countries are becoming aware of the value of information and computer technology in increasing the effectiveness of healthcare delivery [28, 29]. They therefore recommend for their patients to access these sites for further information on their condition. McHugh and colleagues [30] demonstrated that BC is the most commonly researched cancer online. Another study [26] shows that there is a large amount of information on BC available on the Internet for consumers. Many studies have been conducted to evaluate the quality of various websites that provide information on BC to consumers [24–26, 31, 32]. These studies were conducted on websites from developed countries excluding African websites. Moreover, accessing health-related information on the Internet among African women is on the increase due to advances in technology. The current upsurge in the use of mobile broadband data and smartphone could further increase access to health information via the Internet. Therefore, it is necessary to evaluate the quality of African websites available to African women despite them being in their infancy. The purpose of the study is to appraise the websites of both governmental and nonprofit nongovernmental organizations providing BC information to women in Africa. The quality of information would be appraised in terms of completeness, transparency, and accessibility.

## 2. Methodology

### 2.1. Study Design

This study appraised websites run by organizations in African countries that produce cancer information. For the purpose of this study, African countries included were Burundi, Kenya, Rwanda, Tanzania, and Uganda from East Africa, Ghana and Nigeria, West Africa, and South Africa and Zimbabwe. The study was conducted from May to July 2016. The author used a tool developed by Ream and colleagues in 2009 based on suggestions and recommendations from other studies [17, 24, 27] and used previously to investigate the quality of BC information provided on the Internet by voluntary organizations in Great Britain. The tool comprised three sections that scored the completeness, transparency, and accessibility of BC information. These sections had subtopics that were used to appraise the websites.

### 2.2. Sampling of Websites

A convenience sampling method was adopted to obtain the sites to be evaluated. Three searches were conducted using Google search engine (https://www.google.com) and Microsoft search engine (http://www.bing.com) from 15th to 25th May 2016 to generate a list of websites. Other search engines like Ask.com and Quora.com were also used but the results were not different. Other national association websites were also hand-searched. The key words “cancer”; “breast cancer”; and “breast cancer organisations in Africa” were used. The first 100 resultant links and universal resource locators (URLs) of each keyword were reviewed. The criteria for a website to be included were to be owned by African organization and providing information on BC including risk factors, diagnosis, treatments, and psychological support.

Websites were excluded if they were owned by organizations from countries outside Africa, contained only chat rooms or discussion groups, and belonged to a BC charity but did not incorporate any information on BC such as risk factors, diagnosis, treatments, and psychological support on their sites.

### 2.3. Evaluation of the Websites

A detailed evaluation tool developed by Ream et al. [25] to appraise websites was used. The evaluation tool was developed specifically to appraise BC websites based on the European Commission (EC) quality criteria for health-related websites which say that health-related websites should cover the following features in detail, transparency and honesty, authority, privacy and date protection, updating of information, accountability, and accessibility.

This tool was selected because it was inappropriate to use generic evaluation tools for sites providing condition-specific information, as they may not be detailed enough. The evaluation tool developed for appraising the websites covered the following.

*Completeness*. Under this feature, the breadth and depth of various topics relating to BC covered on the websites were appraised. The ten topics included the following: risk factors, screening and mammography, surgical treatment, chemotherapy, radiotherapy, hormonal treatment, other pharmaceutical treatments, breast reconstruction, complementary medicine, and emotional/psychological support. The number of topics covered by the websites (out of maximum of 10) was calculated. After this, the depth of information provided was evaluated. Depth of coverage was clarified for each topic as “no information”; “minimal information”; or “more than minimal information.” In the case of “no information,” the topic under examination was not mentioned at all. “Minimal information” involved a superficial discussion of the topic without provision of detailed information (e.g., surgical treatment might be mentioned as one of the treatments for BC, but with no details on the different types of surgery performed or their implications). A clarification of “more than minimal information” required a detailed discussion of the topic. 

*Transparency*. The subtopics (authorship, attribution, currency, and disclosure) under transparency were formulated based on the EC quality criteria (transparency and honesty, authority, privacy and protection, and updating of information). 

*Authorship*. Authors and contributors, their affiliations, and relevant credentials and contact details should be provided. The status of the author being healthcare provider or expert on BC should be stated. 

*Attribution*. References and sources for all content should be listed clearly, and relevant working external links leading to scientific reference materials should be provided. 

*Disclosure*. Sponsorship, commercial funding arrangements or support, or potential conflicts of interests should be stated. Privacy policy should be fully disclosed and easy to locate. Advertisement on the site was also considered under disclosure.

*Currency*. Dates that content was posted and updated should be clearly indicated. 

Transparency was characterised with “yes for all”; “yes for some”; and “no.” 

*Accessibility*. This related to the websites ease of navigation. The tool determined the ease with which the user could navigate through sites and access their information on BC while maintaining simplicity of technology, operation, or format. Also it assessed whether the site combined text, audio, and visual format to make it accessible to everyone. Presentation and legibility of text as well as colour coordination were part of accessibility that were scrutinised. Accessibility was characterised as “yes always”; “yes sometimes”; and “no.”

### 2.4. Data Analysis

Data under completeness, transparency, and accessibility were analysed and presented in charts (bar chart) and tables showing the median and range for the various data. A summary of the organizations (general cancer and BC specific) was generated for comparison. Scores for topics under transparency (authorship, attribution, currency, and disclosure) were calculated.

## 3. Results

The search generated 25 relevant African sites, both governmental and nonprofit, nongovernmental organizations. However, 12 were excluded because they did not incorporate any information on BC on the sites. Of the remaining 13 websites, five (5) provided information about cancer in general and the remaining five websites were dedicated to creating awareness specifically in relation to BC (Table 1).

General cancer websites comprised 38% of the sample evaluated. Websites from both South Africa (two on general cancer and three BC specific) and Nigeria (two on general cancer and two BC specific) constituted 69% of the websites evaluated, while the remaining 31% were from East Africa (Burundi, Kenya, Tanzania Uganda, and Rwanda), Ghana, and Zimbabwe.

### 3.1. Completeness of Information on Breast Cancer

The completeness of information was evaluated under ten domains for both breadth and depth. Breadth was measuring the number of topics covered by the website. The possible range was 10. Depth entailed measuring the detail in which the topics covered were discussed. The possible range for depth was 20. The total possible range for completeness was 30 with lower score indicating poor performance and higher score indicating high performance.  Table 2 gives details of the breadth and depth of the topics to measure completeness.

All the websites appeared to provide information on screening and mammography and most (11 out of 13) on risk factors. However few (4 out of 13) provided a breadth of information on surgical treatment for BC. While there appeared comprehensive and in-depth information on risk, this appeared not the case for screening and mammography. Most websites provided little more than superficial information on the topic covered. Only 23% gave more than superficial information on emotional and psychological support. None gave more than minimal information on surgical treatment, chemotherapy, radiotherapy, and other pharmaceutical treatments. Two of the websites made mention of other pharmaceutical treatments and only one website which is a BC specific site (http://www.breastcancerafrica.org/) provided information about complementary medicine.

Completeness scores for BC specific sites ranged from 1 to 23 while general cancer sites were from 5 to 13. The overall median score was 6 (range 1 to 23), Table 3. As shown in Table 4, the majority (67%) of the websites scored poorly regarding completeness (score of 0 to 10). The highest score, 23, was scored by a BC specific site (http://www.breastcancerafrica.org/) followed by 20 earned by two BC specific websites (http://www.mybreast.org.za and http://www.reach4recovery.org.za). The lowest score was 1, by a BC specific (http://www.breastcareinternational.org). By comparison, the lowest score attained by a general cancer website was 5.

### 3.2. Transparency

Transparency of information focused on the ease with which the user can identify the author who wrote the web page(s) and their credentials, sources of information, page updates, external scientific links and source of funding. The total possible range of measurement under transparency is 24. Only two (15%) websites (http://www.socron.net and http://www.breastcancer.org) scored 15 each, which is above the average score. The lowest score of 2 was by a BC specific website (http://www.couragetodare.org). The overall median score was 6 (range 2 to 15). This shows that both BC and general cancer websites do not reveal much about the authorship, currency, and attribution of the information being provided, Table 3. Only 2 (15%) of the websites (http://www.cansa.org.za and http://www.pinkdrive.co.za) carried advertisements. When the components for transparency were aggregated it was evident that the majority (76.9%) of the websites scores ranged from 0 to 8 with no scores above 15, Table 4.

Table 5 shows details of scores by various aspects of the transparency score. Most (69%) of the organizations did score zero on authorship. A general cancer organization (http://www.socron.net) scored the highest (8 out of 8) on authorship with one of the BC specific organizations (http://www.breastcancerafrica.org) scoring 5. With regard to attribution, 69% of the organizations failed to disclose the source of information of their material. Only two websites (http://www.breastcancerafrica.org and http://www.cancerbuddies.org.za) scored 4 (out of 7) on attribution, which was the highest score. The median attrition score was 1. Only three websites scored 2 or more on currency, with one general cancer website (http://www.cansa.org.za) scoring 3 (the highest score). Most websites were not updated and did not provide the date of creation of the information given. The majority (77%) of the organizations scored 3 or above on disclosure. A BC specific website (http://www.breastcancerafrica.org) scored 5 (out of the possible range of 5) on disclosure. The lowest score was 2 and the median score was 3.

### 3.3. Accessibility

The final component of websites quality appraisal was accessibility. The overall median score on accessibility was 6 (range 3 to 8). Generally, the overall performance on accessibility was good. Majority, 69.2%, scored between 3 and 7 out of a potential maximum score of 9. Only one of the websites had a score of 3, which was the lowest. The highest score attained was 8 by a general cancer organization website (http://www.socron.net). Only one of the organizations (Society of Oncology and Cancer Research, Nigeria) had information on BC in their local dialect and language other than English language. The website presented information in Yoruba, Hausa, Igbo, and French. Another organization (Breast Health Foundation) also combined text, audio, and visual format to present some of the information on their website.

### 3.4. Overall Performance

The individual domain/aspects of website quality were summed to provide an overall score for evaluation. The potential range of score for overall quality was 63. From Table 4, it can be seen that just over half (53.8%) scored from 0 to 21. Only one of the organizations (Breast Cancer Initiative East Africa, Inc.) scored between 42 and 63 and 38.5% scored from 22 to 41. The overall highest score was 43 recorded by a BC specific organization website (http://www.breastcancerafrica.org). The overall poorest performance was a score of 9, by a BC specific organization website (http://www.couragetodare.org).

**Figure 1:**
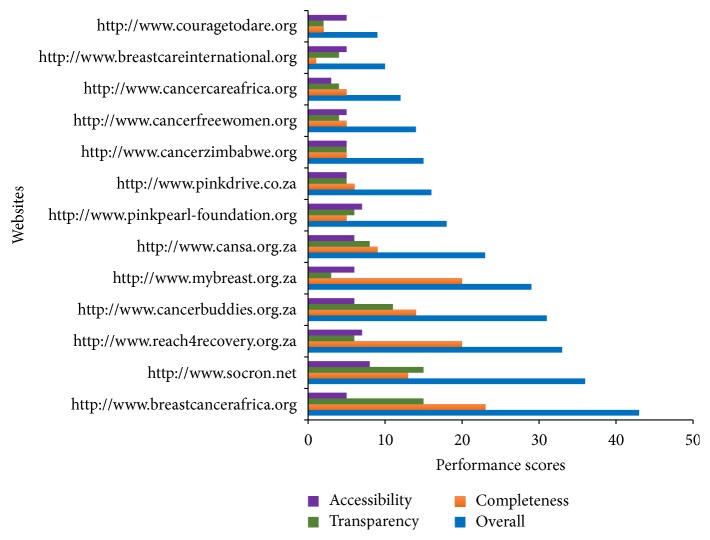
Performance of the websites (completeness, transparency, and accessibility).

From Table 3, the overall median score was 18 (range 9 to 43). The median overall score for BC specific organizations was also 18 (range 9 to 43) and that for general cancer organizations was 17 (12 to 36). From Figure 1, the performance of the websites had been highlighted in a bar chart showing their various scores in terms of completeness, transparency, accessibility, and the overall score. Ten (10) out of the 13 websites studied had less than 50% of the overall performance score.

## 4. Discussion

This study sought to evaluate the information on BC provided on the websites of both governmental and nonprofit nongovernmental organizations in Africa. Their quality was assessed in terms of completeness, transparency, and accessibility. The 13 websites appraised were selected as they were the most common sites identified through systematic searching via Google and Bing search engine purposely to provide information to enhance awareness of BC and its screening and treatment.

Internet access in the African region is usually concentrated in urban and major metropolitan areas, where most of the available bandwidths are located [33]. Most users relied on fixed access through cybercafes for activities such as viewing video or downloading large files [33] and this was the means through which health information was accessed. There is however a current surge in the shift towards wireless Internet in most African countries. Wireless broadband is in the form of paid modem subscriptions, mobile data plans for tablets or smartphones, and free Wi-Fi services offered by educational institutions, businesses, and Internet cafes. This will also have positive influence on access to health information as most people could have access on their mobile phones and tablets. In 2014, smart phones made up 15% of the African mobile market and this is forecasted to rise to 40% by 2017 as smartphones are becoming more affordable [34]. This means that Internet based health information will become more accessible to the populace, therefore the need to constantly appraise the quality to ensure its effective contribution to improving health knowledge of the populace. Although access to health information could improve patients, knowledge, if not properly controlled, will lead to compromised health behaviour and increased anxiety resulting in inappropriate request for health interventions [35].

This study identified that most of the organizations did not provide complete information on BC at their websites. Only two of the 13 websites scored above 20 (out of 30) on completeness. The two lowest scoring websites on completeness were both BC specific websites set up specifically to create awareness in BC. An Internet user visiting these websites may be disappointed and deprived of adequate information as little or superficial information is given on diagnosis and treatment of BC. This was not limited to only the BC specific organizations' websites. The general cancer organizations' websites also lacked detail in both breadth and depth on the topics evaluated.

The mostly covered topic was screening and mammography (all websites covered this topic) followed by risk factors. However, information provided on this was superficial. Only six (out of 13) websites provided more than minimal information on their topics, four of them being BC specific organizations. Complementary medicine was the least covered topic, by only one organization. However, this site provided a good level of details on the topic. It was not surprising the website was a BC specific organization's website and even had the highest score on completeness. The little coverage of complementary medicine on BC websites could be attributed to many recent advances in diagnosis and treatments. These however still remain largely confined to industrialised countries with large disparity existing between the developed and developing countries in terms of research [35]. The websites scoring low on completeness highlight the fact that these organizations do not provide comprehensive information on BC to women. They discussed the topics covered superficially and do not satisfy the consumer's quest for the needed information on BC diagnosis and treatment.

A general cancer organization scored 8 (out of 8) on authorship, followed by a BC specific organization website (5 out of 8). The remaining seven websites did not disclose the names of authors and their credentials as contact details for consumers to know the credibility of the information provided. This study is in accordance with Hoffman-Goetz and Clarke [31] as many of the websites they evaluated lacked information, which would allow the user to assess the credibility of the author of the information, provided. Provision of author's contact details would provide the opportunity to users who seek advice or clarification on any information on BC.

Attribution was assessed based on source of information provided and external links to other scientific materials were the items used to assess attribution. Only one website scored more than half (4 out of 7) under attribution. One-third of the organizations, all BC specific, did not provide any reference to the information provided on their websites. Regardless of the type of organization website, lack of the source of information provided would mask the transparency of the information, contributing to unreliability of the website. External links were not provided by most of the websites especially the BC specific organization websites.

Evaluation of currency was based on provision of date of creation of each page and clarity of recent update. There was a major shortcoming as more than half did not provide any information in order for the currency of the website to be assessed by the user. Some form of information provided by the remaining websites was not clearly stated. Most did not provide information on creation of each page and the last update of the website was not posted. Although posting of recent updates of the information does not guarantee the accuracy of the information, it serves as initial fundamental information that would enable the user to make an informed judgment about the quality of information on the website.

The last item that was assessed under transparency was disclosure which included source of funding of the organization and website, whether the site carried adverts, the clarity of privacy policy, and easiness in its location as well as confidentiality level of the sites. Majority of the websites scored more than half on disclosure and about one-third of the websites clearly stated their source of funding. In general, the websites performed poorly on transparency. This study highlights the need for the websites to provide the identity and credentials of the authors of the information provided. They also need to frequently update the information posted on their sites as it is easy to do so in contrast to traditional print media.

The last component on the evaluation tool was accessibility. It assessed websites features and ease of navigation. Most of the websites scored more than half of the total score. Almost all the websites facilitated navigation through large quantities of information while maintaining simplicity of technology, operation, and format. The websites have logical organization of information and features which speed up finding the location of information by the user. Only two of the websites (all general cancer organizations' websites) provided useful links between sites to help locate specific information. Among all the websites, only one (http://www.socron.net) provided BC information in language other than English. It presented the information in French, Hausa, Igbo, and Yoruba. This feature is important as all African countries have many local dialects being taught and written in schools and so could be read by many who cannot read and write in English language. One website (Breast Health Foundation) combined text, audio, and visual formats to portray information. This makes information more accessible. All the websites use different colours to coordinate between the texts. Also there was lack of clutter of the texts and the information was legible to read. The medians for both BC specific and general cancer organization show that the websites performed better on accessibility than on completeness and transparency. Although majority scored above half out of the possible score, the variations in the score calls for the organizations to make their websites more accessible to users, importantly providing information in different formats, such as large print, audio, videos, and different language/dialect to ensure that BC information on websites is accessible to all specifically the target audience or country. Recognizing the level of health illiteracy in these settings, it is important to consider such features that are appealing and also make it possible and easier to read and appreciate the HI provided.

On the whole, only 3 out of the 13 websites studied had more than 50% of the overall performance score. This indicates the level of incompleteness and lack of detail of information on BC provided by websites in Africa. The finding of this study is consistent with earlier studies that have evaluated the quality of websites [24, 25, 31]. Many websites do not present BC information that gives acceptable, in-depth, and correct coverage as well as lacking even the fundamental information that would enable the user to make an informed judgment about the quality of the website. The WWW or Internet is increasingly becoming popular as a source of health information to consumers especially women on BC issues. Women being abreast with information regarding their disease process are equipped with the information needed to make informed decisions regarding their treatments. Women need accurate and relevant information relating to the disease process including factual data about prognosis, disease stage, potential cure, treatment options, and expected outcome [12, 36]. These are the breadth and depth of the information to be provided by organizations with the purpose of educating women on BC on their website. BC information should be accurate, high in quality, and comprehensive with external scientific links for further information.

The present study documents important findings on the gaps in BC health information provided by websites in Africa. However, there are some limitations. It only gives an account of the situation on the websites included in the present study period. Websites may have changed during and after the period of data collection or ceased to exist. Also new websites may have started up since this study was completed. Information that was actually present on the websites may have been missed and changes that occurred over that period were not considered. Another limitation is that the author (Cynthia Pomaa Akuoko) assessed the quality of the websites alone, which could have introduced some form of bias. Only one search engine was thoroughly used in generating the list of websites; other websites may not have been captured under the one used. The appraisal does not also include some countries in the continent and this may affect the generalizability of the study settings. Finally, the appraisal tool used in this study, also validated and used in other settings, did not capture other important information such as speed of access.

This study recommends that ministries and department of health of various African countries should devise criteria in posting health information on websites to be followed by both governmental and nonprofit nongovernmental organizations in terms of completeness, transparency, and accessibility. This would serve as a guide to anyone, even without any real competence in BC, providing such a website. Moreover, a unified grading or evaluation system, which would allow Internet users to assess these websites, is recommended. This would also allow healthcare providers to guide patients to trustworthy, up-to-date websites, ensuring patients receive high quality information to help them make informed decisions regarding treatment and care. The result shows that there is still a lot of work to be done before African based organizations' websites can serve both professionals and laypersons as valuable, reliable, and up-to-date sources of the latest information on BC health issues.

## 5. Conclusion

The World Wide Web or Internet is increasingly becoming popular as a source of health information to consumers especially women on BC issues. This review has provided evidence of inadequate and inaccurate BC information provided by some cancer organizations in Africa. Considerable effort is required in making BC information on the Internet a valuable and up-to-date source for both professionals and patients.

## Figures and Tables

**Table 1:** Characteristics of included websites.

Organization	Website address	Country
*General Cancer *(*n* = 5)		
Cancer Association of South Africa	http://www.cansa.org.za	South Africa
Cancer Buddies	http://www.cancerbuddies.org.za	South Africa
Cancer Association of Zimbabwe	http://www.cancerzimbabwe.org	Zimbabwe
Cancer Care Africa	http://www.cancercareafrica.org	Nigeria
Society of Oncology and Cancer Research	http://www.socron.net	Nigeria

*Breast Cancer specific *(*n* = 8)		
Breast Cancer Initiative East Africa, Inc.	http://www.breastcancerafrica.org	Kenya, Uganda, Tanzania, Rwanda, Burundi
Breast Health Foundation	http://www.mybreast.org.za	South Africa
Pink Drive	http://www.pinkdrive.co.za	South Africa
Reach for Recovery	http://www.reach4recovery.org.za	South Africa
Pink Pearl Foundation	http://www.pinkpearl-foundation.org	Nigeria
Courage To Dare	http://www.couragetodare.org	Nigeria
Cancer Free Women	http://www.cancerfreewomen.org	Kenya
Breast Care International	http://www.breastcareinternational.org	Ghana

**Table 2:** Fulfilment of completeness criteria.

Topics	Breadth (*n*)	Depth (*n*)		
		No information	Minimal information	More than minimal information
Risk factors	11	2	1	10
Screening & mammography	13	2	5	6
Surgical treatment	4	9	2	2
Chemotherapy	4	9	2	2
Radiotherapy	4	9	2	2
Hormonal treatment	4	9	2	2
Other pharmaceutical Treatments	2	11	1	1
Breast construction	2	11	1	1
Complementary medicine	1	12	0	1
Emotional/psychological support	5	6	2	3

**Table 3:** 

Category of information	Breast cancer specific	General cancer	Overall
	Median (range)	Median (range)	Median (range)
Completeness	6.5 (1–23)	7 (5–14)	6 (1–23)
Transparency	6 (2–15)	7 (4–15)	6 (2–15)
Accessibility	6 (5–7)	6 (6–8)	6 (3–8)
Overall quality score	18 (9–43)	17 (12–36)	18 (9–43)

**Table 4:** Details of breast cancer information.

Category of information	Frequency (*N* = 13)	Percentage
Completeness		
(i) 0–10	8	61.5
(ii) 11–20	4	30.8
(iii) 21–30	1	7.7
Transparency		
(i) 0–8	10	76.9
(ii) 9–17	3	23.1
(iii) 18–24	0	0.0
Accessibility		
(i) 0–3	1	7.7
(ii) 4–6	9	69.2
(iii) 7–9	3	23.1
Overall rating		
(i) 0–21	7	53.8
(ii) 22–41	5	38.5
(iii) 42–63	1	7.7

**Table 5:** Description of transparency categories score.

Category	Median	Range	Possible range
Authorship	0	0–8	0–8
Attribution	1	0–4	0–7
Currency	0	0–3	0–4
Disclosure	3	2–5	0–5
